# The Abortion Web Ecosystem: Cross-Sectional Analysis of Trustworthiness and Bias

**DOI:** 10.2196/20619

**Published:** 2020-10-26

**Authors:** Leo Han, Emily R Boniface, Lisa Yin Han, Jonathan Albright, Nora Doty, Blair G Darney

**Affiliations:** 1 Department of Obstetrics and Gynecology Oregon Health and Science University Portland, OR United States; 2 Department of English Arizona State University Tempe, AZ United States; 3 Department of Film and Media Studies University of California–Santa Barbara Santa Barbara, CA United States; 4 Tow Center for Digital Journalism Graduate School of Journalism Columbia University New York, NY United States; 5 Jersey Shore University Medical Center Department of Obstetrics and Gynecology Neptune, NJ United States; 6 Center for Population Health Research National Institute of Public Health Cuernavaca Mexico; 7 School of Public Health Oregon Health and Science University–Portland State University Portland, OR United States

**Keywords:** internet, abortion, media, websites, infodemiology, infodemic, quality of health information, bias in patient education

## Abstract

**Background:**

People use the internet as a primary source for learning about medical procedures and their associated safety profiles and risks. Although abortion is one of the most common procedures worldwide among women in their reproductive years, it is controversial and highly politicized. Substantial scientific evidence demonstrates that abortion is safe and does not increase a woman’s future risk for depressive disorders or infertility. The extent to which information found on the internet reflects these medical facts in a trustworthy and unbiased manner is not known.

**Objective:**

The purpose of this study was to collate and describe the trustworthiness and political slant or bias of web-based information about abortion safety and risks of depression and infertility following abortion.

**Methods:**

We performed a cross-sectional study of internet websites using 3 search topics: (1) is abortion safe?, (2) does abortion cause depression?, and (3) does abortion cause infertility? We used the Google Adwords tool to identify the search terms most associated with those topics and Google’s search engine to generate databases of websites related to each topic. We then classified and rated each website in terms of content slant (pro-choice, neutral, anti-choice), clarity of slant (obvious, in-between, or difficult/can’t tell), trustworthiness (rating scale of 1-5, 5=most trustworthy), type (forum, feature, scholarly article, resource page, news article, blog, or video), and top-level domain (.com, .net, .org, .edu, .gov, or international domain). We compared website characteristics by search topic (safety, depression, or infertility) using bivariate tests. We summarized trustworthiness using the median and IQR, and we used box-and-whisker plots to visually compare trustworthiness by slant and domain type.

**Results:**

Our search methods yielded a total of 111, 120, and 85 unique sites for safety, depression, and infertility, respectively. Of all the sites (n=316), 57.3% (181/316) were neutral, 35.4% (112/316) were anti-choice, and 7.3% (23/316) were pro-choice. The median trustworthiness score was 2.7 (IQR 1.7-3.7), which did not differ significantly across topics (*P*=.409). Anti-choice sites were less trustworthy (median score 1.3, IQR 1.0-1.7) than neutral (median score 3.3, IQR 2.7-4.0) and pro-choice (median score 3.7, IQR 3.3-4.3) sites. Anti-choice sites were also more likely to have slant clarity that was “difficult to tell” (41/112, 36.6%) compared with neutral (25/181, 13.8%) or pro-choice (4/23, 17.4%; *P*<.001) sites. A negative search term used for the topic of safety (eg, “risks”) produced sites with lower trustworthiness scores than search terms with the word “safety” (median score 1.7 versus 3.7, respectively; *P*<.001).

**Conclusions:**

People seeking information about the safety and potential risks of abortion are likely to encounter a substantial amount of untrustworthy and slanted/biased abortion information. Anti-choice sites are prevalent, often difficult to identify as anti-choice, and less trustworthy than neutral or pro-choice sites. Web searches may lead the public to believe abortion is riskier than it is.

## Introduction

The internet is the first source the public turns to for medical information [1-3]. At least 70% of adults who use the internet use it for health information research, with 43% of them seeking information about specific treatments or procedures [4]. Although the internet is a vast repository of searchable information, there is often incorrect, deliberately misleading, conflicting, and/or hard to understand information [5]. Furthermore, public perception and knowledge is shaped by personalized internet experience, which is delimited by technology company algorithms [6-8]. Abortion is one of the most common medical procedures in the world. According to recent estimates, over 800,000 women in the United States choose an abortion each year [9]; even more consider abortion but do not obtain one [10]. Surgical procedures for abortion are referred to as aspiration, dilation, and curettage (D&C), or dilation and evacuation (D&E) if done later in pregnancy. For abortions in the first trimester of pregnancy (up to 10 weeks), medications are used [11]. The best scientific evidence clearly demonstrates that induced abortion is safe [12,13] and that abortion does not increase a woman’s future risk for disorders such as depression, anxiety, or suicidality [14], or secondary infertility [15,16]. Only low-quality and/or discredited studies suggest otherwise [17,18]. The risks of abortion do increase with the gestational age of the pregnancy, but abortion at any gestational age is safer than childbirth [12].

Despite these facts, many antiabortion arguments rest on misinformation regarding the safety and health consequences of abortion [19]. Crisis pregnancy centers (CPCs)—organizations that try to intercept women who are considering an abortion—often describe abortion as dangerous or deadly in order to dissuade women from choosing to obtain an abortion [20]. In addition, false or misleading information on the internet about abortion continues to exert an influence on public debates and policy [21]. Previous studies have examined web-based abortion information, focusing on the quality of information available for self-referral (on CPC websites, specifically) and about D&E procedures [20,22,23]. These studies found frequent inaccuracies and a lack of comprehensive information about abortion on the internet. However, the studies were limited by small data samples [23,24] or narrow inquiries on specific websites [10]. It remains unclear exactly what a search about abortion safety or potential subsequent risks would yield in terms of websites, as well as the trustworthiness and accuracy of information contained on them. The purpose of this study was to collate and describe the web-based ecosystem about abortion in terms of the trustworthiness and bias about abortion safety and risks. We focused on websites that provided information related to three questions: (1) is abortion safe?, (2) does abortion cause depression?, and (3) does abortion cause infertility? We chose safety, infertility, and depression for our case studies because the existing medical evidence is very clear; the mainstream scientific consensus is that abortion is safe and does not cause subsequent infertility or depression [25]. However, these questions continue to be disputed in public discourse and provide an opportunity to evaluate the quality of website information.

## Methods

This cross-sectional study had two phases: first, we created a database of websites from searches about abortion and safety, depression, and infertility; second, we used the ecosystem database that we created to classify websites in terms of content trustworthiness, political slant on abortion, type of site, and top-level domain.

### Website Database

**Figure 1:**
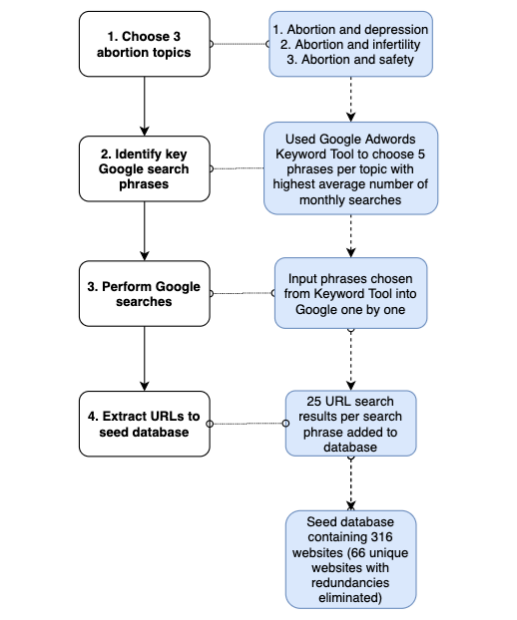
Workflow of website selection for the analysis.

**Figure 2:**
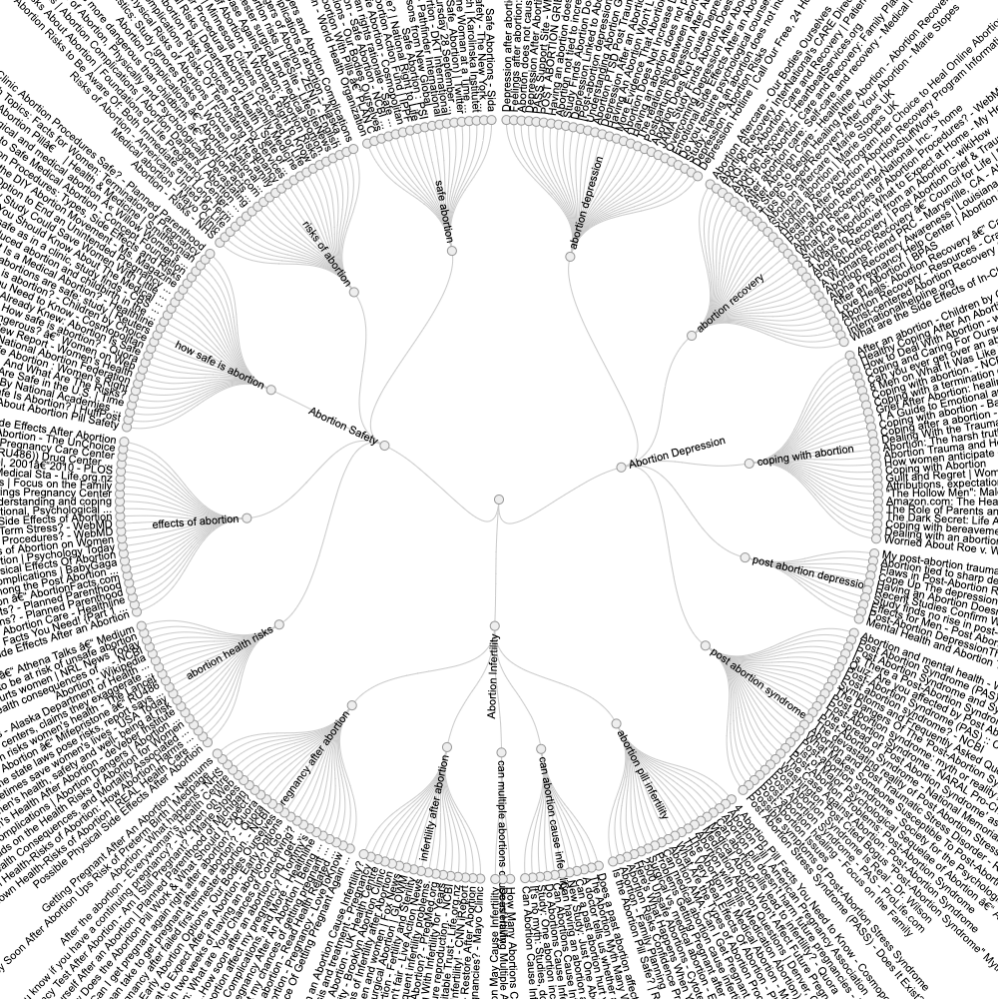
Circular dendrogram of unique websites found in the analysis. The center depicts the 3 keywords used to identify the most commonly searched terms for those topics, the middle ring includes the search phrases that were used for the Google search, and the outer ring depicts the URL search results for each search phrase.

We utilized several steps to create our website database in order to minimize bias and most broadly capture what the general public would encounter while searching on the web (Figure 1). First, we used Google’s Adwords tool, a free Google advertising tool that allows advertisers to associate their ads with search terms based on the popularity of the term. For a given topic, the keyword tool identifies the most commonly used search terms with a topic based on historical search data [26]. We selected 5 search terms for each of our 3 keyword combinations (abortion safety, abortion depression, and abortion infertility) based on their frequency as identified by Google Adwords and relevance (Figure 2). Next, we conducted Google searches using these phrases on August 31, 2018, and compiled website results for each of our search terms. In order to remove search term personalization, each search was performed using a cookie-cleared Google Chrome (version 10.8) browser in incognito mode combined with a free virtual private network called Windscribe (version 1.82) in order to reduce locational bias as well as anonymize our internet protocol addresses. For each search phrase, we extracted the top 25 page-ranked results, noting multiple occurrences. Each link was then imported into a spreadsheet using MozBar (Moz Inc), a Chrome extension that also produced titles, brief excerpts, and a search position number for each link based on its proximity to the top of the Google page. We produced a database for each of our topics (safety, depression, infertility) based on this search methodology.

### Website Categorization

We next classified and rated each website in the database. We rated websites on 5 metrics: (1) slant, (2) slant clarity, (3) trustworthiness, (4) type, and (5) domain. For slant, a website was determined to have a pro-choice or anti-choice bias if either (a) information was given in a biased or dramatic fashion (eg, an anti-choice site describing the procedure as “tearing the baby from the womb”), or (b) the website displayed an opinion regarding the provision of abortion and its legality (eg, a pro-choice site stating that abortion should be free and legal for all women); otherwise, the website was considered neutral. We assigned a slant clarity rating based on how easy it was to discern the website’s slant (obvious, in-between, or difficult/can’t tell). We scored trustworthiness on a rating scale from 1 to 5, with 5 being most trustworthy based on factors including the content and the source of the content (eg, creators, references, or affiliation with health care organizations or health care professionals) [27-29]. These factors were informed by existing systematic criteria developed by prior studies of media credibility and disinformation [30]. The domain was the generic top-level domain of the site (.com/.net, .edu, .gov, .org, or a domain that identified the website as international, such as .uk or .nz). Finally, investigators loosely categorized websites based on 7 descriptive “types,” including forum, feature, scholarly article, resource page, news article, blog, and video. Two investigators independently coded all of the websites. In the event of disagreement, a third researcher arbitrated the categorization. In the case of trustworthiness, the scores from investigators were averaged. All three researchers have published abortion-related research previously.

### Data Analysis

First, we created a visualization of the websites and their parent search terms and topics using RAWGraphs. Next, we compared website characteristics overall and by search topic (safety, depression, or infertility) using Pearson chi-square test or Fisher exact test for all categorical classifications to compare characteristics across topics. We summarized trustworthiness, measured on a 5-point scale, using the median and IQR. For each search topic, we also assessed the distribution of websites based on the content’s slant for the 5 query terms, and then compared the trustworthiness of query term results using Kruskal-Wallis and Wilcoxon rank sum tests. We used box-and-whisker plots to visually compare trustworthiness by slant and by domain type. Finally, we tabulated websites according to their slant and slant clarity, comparing distributions using Fisher exact test, and then used a bar graph to examine website counts graphically. The study was reviewed and approved by the Oregon Health and Science University’s institutional review board and was deemed not human subjects research.

## Results

After removing duplicates, our search methods yielded a total of 111, 120, and 85 unique sites for safety, depression, and infertility, respectively. Figure 2 provides a visualization of these websites and their parent search terms and topic (see Multimedia Appendix 1 for full list of sites with URLs). While the majority of sites had a neutral slant (181/316, 57.3%) overall, the slant distribution differed by topic, with a higher proportion of sites about safety and depression having an anti-choice slant (Table 1). Approximately 40% of sites about safety and depression had an anti-choice slant (safety: 44/111, 39.6%; depression: 51/120, 42.5%), compared with 15.3% (17/111) and 3.3% (4/120) of the safety and depression sites, respectively, being categorized as having a pro-choice slant. Infertility had the highest proportion of neutral sites (66/85, 77.7%). In terms of website type, we categorized the majority of websites as resource pages (158/316, 50.8%). Commercial (.com/.net) (154/316, 48.7%) and organization (.org) (101/316, 32.0%) sites accounted for the majority of domain types. While we did not specifically perform an analysis of slant by web address (URL), we noticed that many of the anti-choice sites came from generic-appearing addresses (eg, americanpregnancy.org, adviceandaid.com). One state health department site (Alaska) was rated by our researchers as being anti-choice.

**Figure 3:**
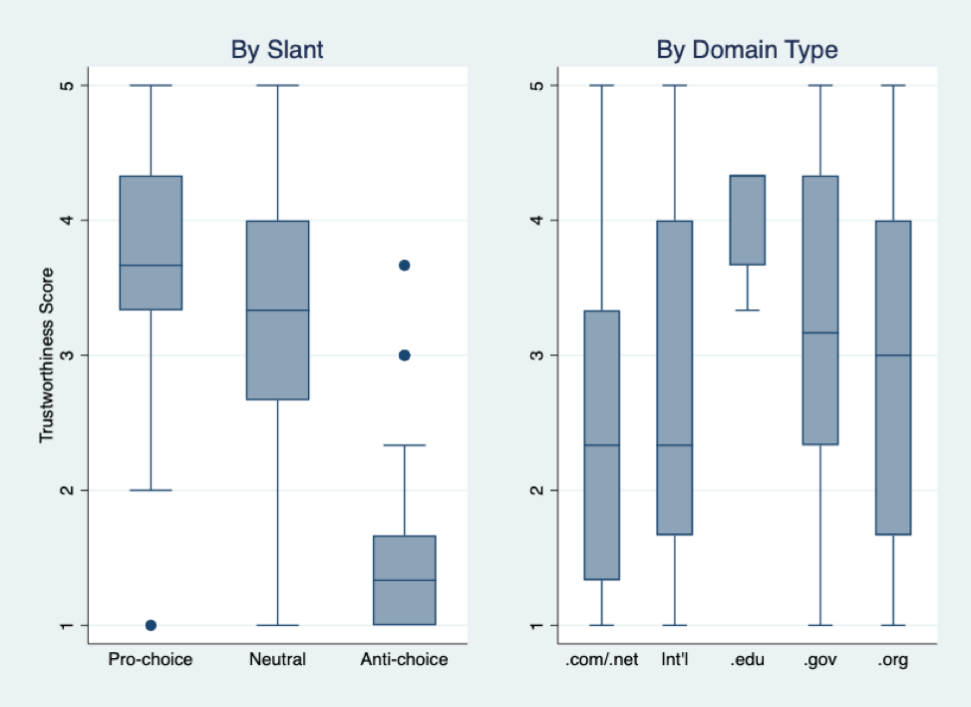
Box-and-whisker plots comparing trustworthiness of online abortion resources by slant and domain type (n=316). Trustworthiness was scored on a scale of 1 (least trustworthy) to 5 (most trustworthy). The central line in each box marks the median trustworthiness score; upper and lower box edges mark the 75th and 25th percentile, respectively; whiskers indicate 150% of the interquartile range and outliers are shown as individual points. Int’l: international domain (eg, .uk, .nz, .id).

We found that the median score for trustworthiness of the websites was 2.7 (IQR 1.7-3.7), which did not differ significantly across topics (safety: median score 3.0, IQR 1.7-3.7; depression: median score 2.3, IQR 1.7-3.7; infertility: median score 2.7, IQR 1.7-3.7; *P*=.409). Agreement in trustworthiness scores among our coders was 71.8%, 70.0%, and 80.9% for safety, depression, and infertility, respectively. Of the 316 sites in our sample, only 59 (18.7%) sites received median trustworthiness scores of 4 or more (data not shown). The trustworthiness rating of sites was different by slant and by domain type (Figure 3). Overall, anti-choice sites had lower trustworthiness scores (median score 1.3, IQR 1.0-1.7) compared with neutral (median score 3.3, IQR 2.7-4.0) and pro-choice (median score 3.7, IQR 3.3-4.3) sites. Sites that came from educational institutions (.edu) were consistently given higher trustworthiness scores (median score 4.3, IQR 3.7-4.3), while other domains received overall lower and more widely distributed trustworthiness scores, with commercial sites (.com/.net) considered to be the least trustworthy (median score 2.3, IQR 1.3-3.3).

**Table 1:** Website characteristics by search topic (n=316).

Characteristic		Overall		Safety (n=111)^a^		Depression (n=120)		Infertility (n=85)		*P* value	
**Slant, n (%)**											<.001
	Pro-choice	23 (7.3)		17 (15.3)		4 (3.3)		2 (2.4)			
	Neutral	181 (57.3)		50 (45.1)		65 (54.2)		66 (77.7)			
	Anti-choice	112 (35.4)		44 (39.6)		51 (42.5)		17 (20.0)			
**Slant clarity, n (%)**											.002
	Obvious	121 (38.3)		41 (36.9)		49 (40.8)		31 (36.5)			
	In-between	125 (39.6)		49 (44.1)		33 (27.5)		43 (50.6)			
	Difficult/can’t tell	70 (22.1)		21 (18.9)		38 (31.7)		11 (12.9)			
**Domain, n (%)**											.002
	Commercial (.com/.net)	154 (48.7)		45 (40.5)		60 (50.0)		49 (57.7)			
	International	42 (13.3)		8 (7.2)		17 (14.2)		17 (20.0)			
	Education (.edu)	5 (1.6)		2 (1.8)		1 (0.8)		2 (2.4)			
	Government (.gov)	14 (4.4)		8 (7.2)		4 (3.3)		2 (2.4)			
	Organization (.org)	101 (32.0)		48 (43.2)		38 (31.7)		15 (17.7)			
**Page type, n (%)**											.007
	News article	30 (9.7)		12 (11.3)		13 (10.8)		5 (5.9)			
	Forum	17 (5.5)		2 (1.9)		2 (1.7)		13 (15.3)			
	Blog	16 (5.1)		5 (4.7)		8 (6.7)		3 (3.5)			
	Feature	63 (20.3)		21 (19.8)		20 (16.7)		22 (25.9)			
	Scholarly article	25 (8.0)		9 (8.5)		11 (9.2)		5 (5.9)			
	Resource page	158 (50.8)		57 (53.8)		64 (53.3)		37 (43.5)			
	Video	2 (0.6)		0 (0)		2 (1.7)		0 (0)			
Trustworthiness, median (IQR)			2.7 (1.7-3.7)		3.0 (1.7-3.7)		2.3 (1.7-3.7)		2.7 (1.7-3.7)		.409

^a^Five websites in the safety category were missing data about page type.

We also examined our results using specific search query terms and noted that for searches on safety and infertility, trustworthiness scores were significantly different across query search terms (Table 2). For searches focused on abortion safety, including the word “safe” resulted in sites with significantly higher trustworthiness scores (median score 3.7, IQR 3.3-4.3) compared with searches that included the more negative word “risks” (median 1.7, IQR 1.0-3.3; Wilcoxon rank sum *P*<.001). Anti-choice sites appeared more frequently with negative search terms, such as “risks of abortion” (17/23, 73.9%), compared with when the word “safe” was included in the search term, such as, “How safe is abortion?” (1/27, 3.7%) (Table 2).

**Figure 4:**
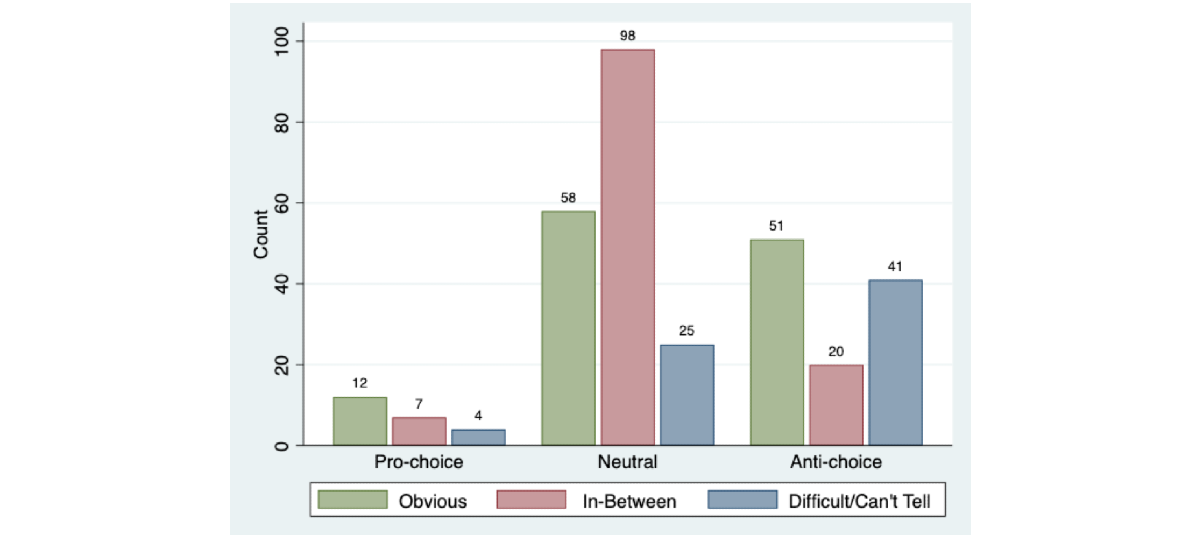
Counts of online abortion resources by slant and slant clarity (n=316).

Finally, we found that slant clarity, or how difficult it was to discern the anti-choice, neutral, or pro-choice slant of a website, varied significantly both by topic and by slant. In regard to the topic, there were many more depression websites with a “difficult/can’t tell” slant compared with sites about safety and infertility (31.7% versus 18.9% and 12.9%, respectively; *P*=.002). However, all 3 topic categories contained clarity ratings of “obvious” for fewer than one-half of the sites (Table 1). In terms of slant, anti-choice sites were more likely to have slant clarity that was “difficult/can’t tell” (41/112, 36.6%) compared with neutral (25/181, 13.8%) or pro-choice (4/23, 17.4%) sites (*P*<.001) (Figure 4).

**Table 2:** Search query terms used and associated trustworthiness and website slant for each search topic.

Topic and search query terms		Total, n (%)	Pro-choice, n (%)	Neutral, n (%)		Anti-choice, n (%)	Trustworthiness, median (IQR)		*P* value
**Safety (n=111)**									<.001
	Abortion health risks	19 (17.1)	1 (5.3)		7 (36.8)	11 (57.9)		2.3 (1.7-3.3)	
	Effects of abortion	24 (21.6)	0 (0)		10 (41.7)	14 (58.3)		1.7 (1.0-3.0)	
	How safe is abortion?	27 (24.4)	5 (18.5)		21 (77.8)	1 (3.7)		3.7 (3.0-4.3)	
	Risks of abortion	23 (20.7)	0 (0)		6 (26.1)	17 (73.9)		1.3 (1.0-2.3)	
	Safe abortion	18 (16.2)	11 (61.1)		6 (33.3)	1 (5.6)		3.7 (3.7-4.3)	
**Depression (n=120)**									.143
	Abortion depression	28 (23.3)	0 (0)		17 (60.7)	11 (39.3)		3.2 (1.7-3.7)	
	Abortion recovery	29 (24.2)	0 (0)		14 (48.3)	15 (51.7)		2.3 (1.7-3.7)	
	Coping with abortion	26 (21.7)	0 (0)		21 (80.8)	5 (19.2)		3.2 (1.7-3.7)	
	Post-abortion depression	10 (8.3)	0 (0)		6 (60.0)	4 (40.0)		2.2 (1.3-3.3)	
	Post-abortion syndrome	27 (22.5)	4 (14.8)		7 (25.9)	16 (59.3)		1.7 (1.3-3.0)	
**Infertility (n=85)**									.005
	Abortion pill infertility	19 (22.4)	1 (5.3)		15 (78.9)	3 (15.8)		3.7 (2.0-4.3)	
	Can abortion cause infertility?	11 (12.9)	0 (0)		8 (72.7)	3 (27.3)		2.0 (1.7-3.3)	
	Can multiple abortions cause infertility?	15 (17.6)	0 (0)		11 (73.3)	4 (26.7)		1.7 (1.0-2.7)	
	Infertility after abortion	17 (20.0)	1 (5.9)		10 (58.8)	6 (35.3)		2.3 (1.0-3.3)	
	Pregnancy after abortion	23 (27.1)	0 (0)		22 (95.7)	1 (4.3)		3.0 (2.3-3.7)	

## Discussion

### Principal Findings

Our study of websites that provide information about safety of abortion or subsequent risks of depression or infertility found that overall, 35.4% (112/316) of sites had an anti-choice slant. Anti-choice sites were less trustworthy, and the clarity of their stance was also more difficult to determine. Our data also show that people seeking information about the safety and potential risks of abortion are likely to find substantial amounts of untrustworthy abortion information; the overall median trustworthiness score of these sites was 2.7 (on a scale of 1-5), with a wide range (IQR 1.7-3.7).

Our results are consistent with other examples of intentionally disseminated abortion disinformation. CPCs have an antiabortion agenda but use neutral language and advertising to appear as if they are a normal medical clinic [20]. In our study, almost two-thirds (71/112, 63.4%) of anti-choice sites were categorized as resource pages and many derived from web addresses that appeared generic and neutral. Moreover, one state health department site rated as anti-choice, which demonstrates that even official governmental sites can be biased sources of information. These findings suggest that anti-choice sites may be trying to obscure their biases or appear as neutral information brokers. Based on the high number of “difficult to tell” sites in our data sets, we need more research to understand the characteristics of anti-choice sites that create this effect.

We know that in the case of abortion, false or misleading information continues to play a role in public debates and government legislation. For example, Targeted Regulation of Abortion Providers (TRAP) laws, or costly and burdensome regulations aiming to restrict access to abortions by dictating how, by whom, and when abortions can be provided, are often based on false health claims but framed as protecting patient safety [31]. The internet is one of the most important propagators of false information. Previous research has shown that media is more influential among young people (aged 13 to 29 years) than friends, family, and health care providers when it comes to learning about abortion [32]. However, there is also evidence that exposing the public to more evidence-based information on abortion can change opinions about the provision and regulation of abortion services [33]. Thus, efforts to address the spread of false information and strengthen the presence of evidence-based information on abortion can have real impact. Our results can help inform clinicians and others who work in the abortion field about likely information gaps [34]. Moreover, health organizations that seek to disseminate evidence-based information may want to conduct regular search audits such as this in order to optimize their search positions to reach a broader audience.

### Limitations

Our study results should be interpreted with the following limitations in mind. We used expert ratings to gain insight into the abortion online media ecosystem. All of our experts are engaged in abortion-related research and two-thirds of our experts are abortion providers. We recognize that non-experts may have rated and classified these same websites differently. However, our expert ratings provide a benchmark for future work that explores how non-experts perceive the information contained in these websites. We also acknowledge that trustworthiness scores could be confounded with slant and that sites with obvious anti-choice slants may have been rated with lower trustworthiness scores based on the impression of bias. However, it is difficult to blind these components from each other and we wanted to provide some kind of measure of quality for the information we encountered. We recognize that our study provides only a cross-sectional snapshot of the internet. While specific site rankings may be driven by news cycles, we believe the overall picture is unlikely to change [35]. We also acknowledge that while we took measures to anonymize the search results, individuals will receive different results based on their previous search history, and the majority will not scroll past the first page. However, our database sizes captured approximately the first 8 to 12 pages of search results for a given topic. Finally, while our focus was on websites, websites alone do not fully address the digital means through which abortion information is shared. In particular, social networking platforms are critical sources [36]. The strengths of our study include the use of a systematic and rigorous approach to create our website database, incorporation of multiple topic areas, and focus on content trustworthiness and bias.

Our results provide insight into the online abortion ecosystem. We find that anti-choice sites are prevalent, often hard to identify as anti-choice (difficult slant clarity), and less trustworthy than neutral and pro-choice sites. Additionally, the search terms a user chooses may play a substantial role in the quality and bias of websites they see. Our results help us understand how the internet may impact public perceptions and knowledge about the safety of abortion and potential risks of depression and infertility. Our findings suggest that web searches may lead people to perceive abortion procedures as more dangerous and riskier than they actually are.

## Appendix

Multimedia Appendix 1 Full list of websites and site ratings.

## Abbreviations

CPCs: crisis pregnancy centers
D&C: aspiration, dilation, and curettage
D&E: dilation and evacuation
TRAP: Targeted Regulation of Abortion Providers
